# Supplementary material for: The expression of stem cells markers and its effects on the propensity for recurrence and metastasis in bladder cancer: A systematic review
Source: PLoS One. 2023 May 17;18(5):e0269214. doi: 10.1371/journal.pone.0269214 (PMC10191355; doi:10.1371/journal.pone.0269214)
Supplement: Attachment — Submitted filename: Response to Reviewers 2.docx [file pone.0269214.s003.docx]

**Response to Reviewers**

Reviewer #1

Both tables span multiple pages and are difficult to understand at a glance. The tables need to be in the range of one or two pages.

- Thank you for your feedback, we have adjusted the table sizes accordingly whilst maintaining the amount of content within them

Some of the cancer stem cell genes and stem cell genes in the paper are not very familiar. To be sure, all of the genes need to be explained and verified.

- We have included a brief description of all stem cell genes

There is no uniformity in the words used within the tables. For example, is there any difference between qrtRT-PCR, qRT-PCR, and RT-q-PCR? It would be more helpful to the reader if the designations were unified whenever possible.

- The referred designations have been unified. Apologies for the earlier confusion as we tried to stick with the terms the authors used in their respective studies

Some studies do not seem to correlate these stem cell gene expressions with prognostic factors, but the abstract reads as if there were significant differences in all studies.

- Thank you for bringing this to our attention, we have reworded that section on the abstract accordingly

The stem cell significance of bladder cancer should be discussed, as it has been mentioned in the recent TCGA studies (PMID: 24476821, 28988769, 29617660).

- We appreciate this suggestion and have included the significance of stem cell in bladder cancer according to recent TCGA studies

Reviewer #2

1. If the author could validate their finding with another independent cohort of samples to confirm, some of these results will be perfect. For example, according to the association between gene/protein expression and recurrence, analysis of TCGA (PanCancer Atlas) data is able to confirm that the expression of their isolated markers showed poor outcomes in bladder cancer. The authors can freely download and analyze the dataset from cBioportal (<http://cbioportal.org>).

- Thank you for the additional sources of reference, we have incorporated these additional findings to support our discussion

2. “The expression of cancer stem cells” in the title is strange and overestimate. The authors only link the expression of stem cell markers, but not the expression of cancer stem cells, to poor clinical outcome. Please change the title.

- We thank you for bringing this to our attention. The appropriate title changes have been made
