# Supplementary material for: The expression of stem cells markers and its effects on the propensity for recurrence and metastasis in bladder cancer: A systematic review
Source: PLoS One. 2023 May 17;18(5):e0269214. doi: 10.1371/journal.pone.0269214 (PMC10191355; doi:10.1371/journal.pone.0269214)
Supplement: Attachment — Submitted filename: Response to Reviewers 3 and 4.docx [file pone.0269214.s004.docx]

**Response to Reviewers**

Reviewer #3

1. Are there any other potential stem cell genes related to the prognosis of BCa?

- Dear reviewer, as of the writing of this paper all the identified stem cell genes that may be related to the prognosis of BCa have been included in this writing

2. Do the prognoses of these stem cell genes have any difference in all and subtypes of BCa patients?

- Dear reviewer, to our knowledge no study has yet been performed on the effects of individual stem cell genes on each subtype of BCa patients

3. Please check the format of reference in the whole paper. For example, full stop in line 49 “…metastasize. [1-3] However, both…” is before or after [1-3]?

- We place the reference numbers after full stops

4. When author illustrated the data from table 3, they mentioned 13 stem cell genes in line 152-154. But why only 9 stem cell genes are shown in table 3?

- Dear author we apologize for the confusion, this is because out of the 13 stem cell genes studied by the identified articles, only 9 of them performed univariate or multivariate analyses to compare in Table 3

5. It’s better to explain the full name of stem cell genes for the first time.

- Thank you for your feedback, we have added the full names of each stem cells accordingly

6. It’s better to state the results in detail.

- Thank you for the feedback we have corrected accordingly

7. In line 178, “…of In …” may be a typo mistake.

- Thank you for the feedback we have corrected accordingly

Reviewer #4

1. The review may describe comprehensive data in the results section.

- We thank you for the feedback and have described the data in greater detail

2. There was no evaluation and analysis to compare different markers of BCSCs in the same outcome group.

- Yes we did not perform further analyses such as forest plots for this article

3. The relationship of these BCSCs makers should be discussed.

- We thank you for the feedback and have added the points accordingly
